# miR-8-3p regulates *mitoferrin* in the testes of *Bactrocera dorsalis* to ensure normal spermatogenesis

**DOI:** 10.1038/srep22565

**Published:** 2016-03-02

**Authors:** Kaleem Tariq, Christoph Metzendorf, Wei Peng, Summar Sohail, Hongyu Zhang

**Affiliations:** 1 State Key Laboratory of Agricultural Microbiology, Institute of Urban and Horticultural Entomology, College of Plant Science and Technology, Huazhong Agricultural University, Wuhan 430070, Hubei, People’s Republic of China; 2 Heidelberg University Biochemistry Center (BZH), University of Heidelberg, ImNeuenheimer Feld 328, Heidelberg, Germany

## Abstract

Genetics-enhanced sterile insect techniques (SIT) are promising novel approaches to control *Bactrocera dorsalis*, the most destructive horticultural pest in East Asia and the Pacific region. To identify novel genetic agents to alter male fertility of *B. dorsalis*, previous studies investigated miRNA expression in testes of *B. dorsalis*. One miRNA, miR-8-3p was predicted to bind the 3′UTR of putative *B. dorsalis mitoferrin* (*bmfrn*). The ortholog of *bmfrn* in *D. melanogaster* is essential for male fertility. Here we show that *bmfrn* has all conserved amino acid residues of known mitoferrins and is most abundantly expressed in *B. dorsalis* testes, making miR-8-3p and *mitoferrin* candidates for genetics-enhanced SIT. Furthermore, using a dual-luciferase reporter system, we show in HeLa cells that miR-8-3p interacts with the 3′UTR of *bmfrn*. Dietary treatments of adult male flies with miR-8-3p mimic, antagomiR, or *bmfrn* dsRNA, altered *mitoferrin* expression in the testes and resulted in reduced male reproductive capacity due to reduced numbers and viability of spermatozoa. We show for the first time that a *mitoferrin* is regulated by a miRNA and we demonstrate miR-8-3p as well as *bmfrn* dsRNA to be promising novel agents that could be used for genetics-enhanced SIT.

The oriental fruit fly, *Bactrocera dorsalis* (Hendel) is one of the most destructive pests of horticultural crops, causing damage to over 250 different types of fruits and vegetables and is widely distributed throughout Southeast Asia and several Pacific Islands^1^. *B. dorsalis* belongs to the family Tephritidae, a family that diverged from Drosophilidae approximately 70 million years ago^2^. Chemical control is currently considered to be the most effective way to combat against fruit flies. However, owing to ever increasing resistance against different insecticides, their negative impact on ecosystems and danger to human health, it will be of great importance to develop new methods to control this pest^3^. Traditional sterile insect techniques (SIT) use chemicals or irradiation to generate sterile insects to be released in target regions at high numbers to compete against their wild counterparts for the respective mating partner. However, irradiation or chemosterilants used for sterilization of male insects decrease their mating performance, which ultimately reduces pest control efficiency^3^. Recently, genetics-enhanced SIT have shown large potential for olive fruit fly management, where classical SIT had failed due to altered mating behavior of the SIT-flies compared to animals in the wild^3^. Also production of sterile male mosquitoes using RNAi-mediated knockdown of male fertility genes has successfully been employed^4^. Agrawal *et al.* introduced a new strategy of delivering genetic agents to phytophagous insects. They generated transgenic tobacco plants, over-expressing insect-specific microRNA amiR-24, resulting in successful knock-down of *chitinase* gene-expression in *Helicoverpa armigera,* leading to lethality in this insect pest^5^.

Spermatogenesis is a complex biological process and strongly regulated on both, the transcriptional and translational level, through temporal and spatial expression patterns of genes^6^. Emerging evidence shows that microRNAs (miRNAs) are indispensable for spermatogenesis of many organisms, including *Drosophila*^7^,^8^,^9^,^10^. miRNAs are small (18–24 nucleotides nt), non-coding RNAs that regulates almost all biological processes in eukaryotes^11^,^12^. In general, miRNA-mediated repression of target mRNAs is a flexible and efficient mechanism that modulates spatio-temporal expression of genes. In *B. dorsalis* it was recently shown that miRNAs are differentially expressed during development^13^.

Previously, we have identified 172 known and 78 novel miRNAs in different developmental stages of *B. dorsalis* testes. Using a bioinformatic approach, we predicted 124 target genes for the 13 most differentially expressed miRNAs^14^. Our bioinformatic analysis suggested that among them, five miRNAs may target the *mitoferrin* gene, *Mitoferrin* belongs to the mitochondrial carrier family (SLC25) and orthologous genes can be identified across a broad range of eukaryotic species^15^. It is involved in iron transport into mitochondria^16^,^17^ and is required for the synthesis of heme and iron-sulfur clusters under iron limiting conditions^18^. *Mitoferrin* has been reported to be indispensable for spermatid elongation and individualization and thus for male fertility in *Drosophila melanogaster*^19^. While sterility of male flies with hypomorph *dmfrn* alleles increased with decreasing dietary iron availability, it was fully penetrant in flies with null alleles of *mitoferrin*. This indicates that mitoferrins function in the mitochondrial iron metabolism^15^,^18^,^20^ is required during spermatogenesis. Therefore, the miRNAs potentially targeting *mitoferrin* mRNA could represent good candidates for the generation of genetics-enhanced SIT approaches for the control of oriental fruit flies.

We report, for the first time, a miRNA targeting *mitoferrin*. We show that dietary delivery of both, miR-8-3p mimics and miR-8-3p antagomiR as well as *B. dorsalis mitoferrin* dsRNA affect *mitoferrin* expression in the testes of *B. dorsalis*. Furthermore, these alterations significantly reduce male fertility, indicating that targeting mitoferrin expression via miRNA, antagomiRs or dsRNA are promising novel agents that may be used to develop non-radiated and non-transgenic SIT techniques to control *B. dorsalis.*

## Results

### miR-8-3p interacts with the *mitoferrin* 3′UTR and represses *mitoferrin* mRNA

In our previous work we have obtained miRNA profiles at different developmental stages during spermatogenesis of the oriental fruit fly^14^. Five miRNAs showed sequence similarity to the 3′-UTR of the putative *mitoferrin* mRNA of *B. dorsalis* testes. As ablation of *mitoferrin* resulted in male sterility in *Drosophila*^19^, we found it a highly interesting candidate gene for genetics-enhanced SIT. We first conducted bioinformatic sequence analysis of the putative *B. dorsalis* mitoferrin protein. We aligned the amino acid sequence of the putative bmfrn to those of known mitoferrins from yeast, *Drosophila* and human. A high degree of sequence conservation between putative bmfrn and known mitoferrins and the presence of the conserved histidine residues (H48, H105 and H222, numbering according to MRS3) responsible for iron transport^16^ argue strongly for bmfrn being the mitoferrin of *B. dorsalis* (Supplementary figure S1).

**Figure 1:**
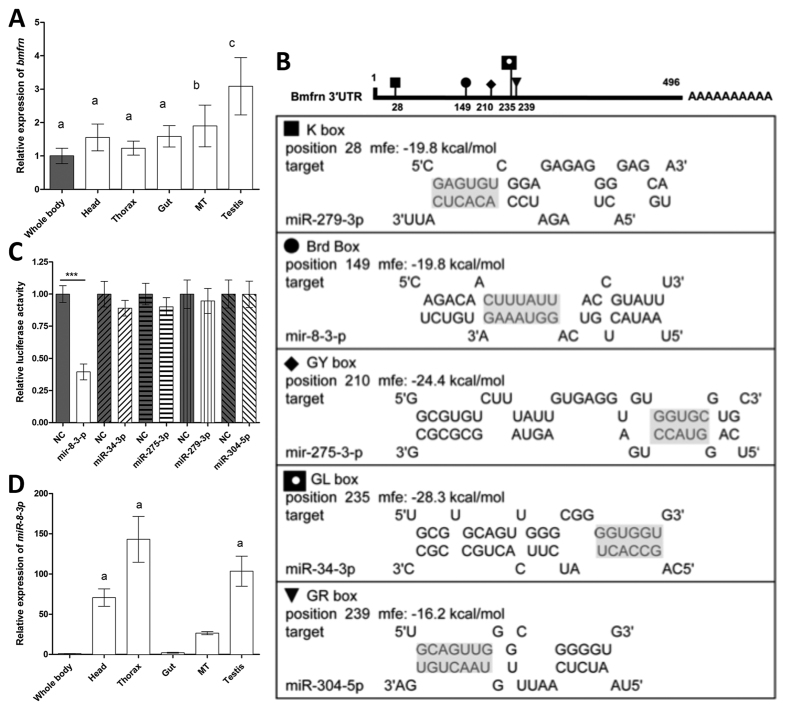
Mitoferrin is most abundantly expressed in testes and targeted by miR-8-3p. (**A**) Expression pattern of the *B. dorsalis* gene *mitoferrin* in different tissues (whole body, head, thorax, gut, malpighian tube (Mt) and testis) of 12 d old adult males (n = 20). *Mitoferrin* expression in tissues is relative to whole body expression. The letters above the bars show significant differences (Least Significant Difference in one-way analysis of variance, *P* < 0.05) in *bmfrn* expression. The data represent the mean of three independent experiments. Error bars indicate SD. (**B**) Potential miRNA target sites of miR-279-3p, miR-8-3-p, miR-275-3p, miR-34-3p and miR-304-5pin the 3′-UTR of the *mitoferrin* as detected by RNAhybrid. Seed sequence of the miRNAs and their putative binding sites in the 3′-UTR are indicated by grey shading. mfe:match free energy. (**C**) Dual-luciferase assay in HeLa cells co-transfected with psiCHECK-2 -*bmfrn* 3′-UTR (100 ng) together with negative control miRNA (miR-NC) or miRNA mimics (50 nM) as indicated. Data represent means of three independent experiments, error bars indicate SD. ****P < 0.001, ANOVA with Bonferroni’s Multiple Comparison Test, testing selected pairs (miRNA vs respective miR-NC). (**D**) Expression pattern of *miR-8-3p* in different tissues (whole body, head, thorax, gut, malpighian tube (Mt) and testis) of 12 d old adult males (n = 3). Expression in tissues is relative to whole body expression. “a” above the bars indicates significant differences in *miR-8-3p* expression compared with whole fly homogenate. The data represent the means with SD. N = 3.

Next we wanted to investigate, whether the tissue specific expression pattern of *bmfrn* were similar to that of the *mitoferrin* gene of *Drosophila*, which is ubiquitously expressed in different tissues but most abundantly in testes^19^. We performed qRT-PCR with cDNA generated from RNA isolated from heads, thoraxes, guts, malpighian tubules and testes of 12 days old virgin male flies and analyzed the expression level of *bmfrn*. We found that *bmfrn,* as *dmfrn* in *Drosophila*^19^, was most abundantly (*P* < 0.001) expressed in testes as compared to heads, thoraxes, guts and malpighian tubules (Fig. 1A).

The bioinformatic analysis of the *bmfrn* together with the tissue expression pattern strongly suggest that mitoferrin in *B. dorsalis* could have a similar function in spermatogenesis as mitoferrin in *D. melanogaster*.

To characterize the interaction between the five miRNAs (miR-279-3p, miR-8-3-p, miR-275-3p, miR-34- 3p and miR-304-5p) and the 3′-UTR of *bmfrn* mRNA, the full length sequence of the 3′-UTR of *bmfrn* and the sequences of all miRNA candidates were submitted online to RNAhybrid, an algorithm taking into account the free energy level of RNA-RNA duplexes and degree of miRNA target sequence complementarity^21^. The 5′ seed sequences of all five candidates were found to be complementary to sites in the 3′-UTR of the *bmfrn* gene. The smallest free energy value was observed formiR-34-3p (Fig. 1B).

To experimentally test, which of these miRNAs affects *bmfrn* expression *in vivo*, we used the dual luciferase assay. To this end a firefly luciferase reporter-construct was made by inserting the 3′-UTR of *bmfrn* downstream of the renilla luciferase coding sequence of thepsiCheck-2 vector. This vector also expresses firefly luciferase allowing signal normalization. To reduce interaction of endogenous miRNAs and mRNAs with our test system, we used HeLa cells for co-transfection with the dual-luciferase reporter and miRNA mimics. Whereas co-transfection with miR-34, miR-275, miR-279 and miR-304 did not result in significantly altered luciferase signal, approximately 2.5 fold reduction in luminescence was observed upon co-transfection with miR-8-3p as compared to controls (Fig. 1C). This suggests that miR-8-3p is the only miRNA, of the five miRNAs tested, that interacts with the 3′ UTR of *mitoferrin* mRNA and could be an interesting candidate to develop genetic SIT to control *B. dorsalis*. Finally, to characterize miR-8-3p expression in more detail, we obtained its tissue expression profile. miR-8-3p was expressed most abundantly in heads, testes and thoraxes and least expressed in whole fly, guts and malpighian tubule (Fig. 1D).

### Dietary delivery of dsRNA, miR-8-3p mimics/antagomiR successfully alters *bmfrn* expression in testes and reduces reproductive capacity of male flies

Previously it was shown that RNAi and miRNA as well as antagomiRs can be successfully delivered to moth larvae and oriental fruit flies via feeding^22^,^23^. This is a very convenient method to manipulate gene expression, allowing us to explore the role of miR-8-3p in spermatogenesis and its applicability as a target for SIT without the need to generate transgenic animals.

**Figure 2:**
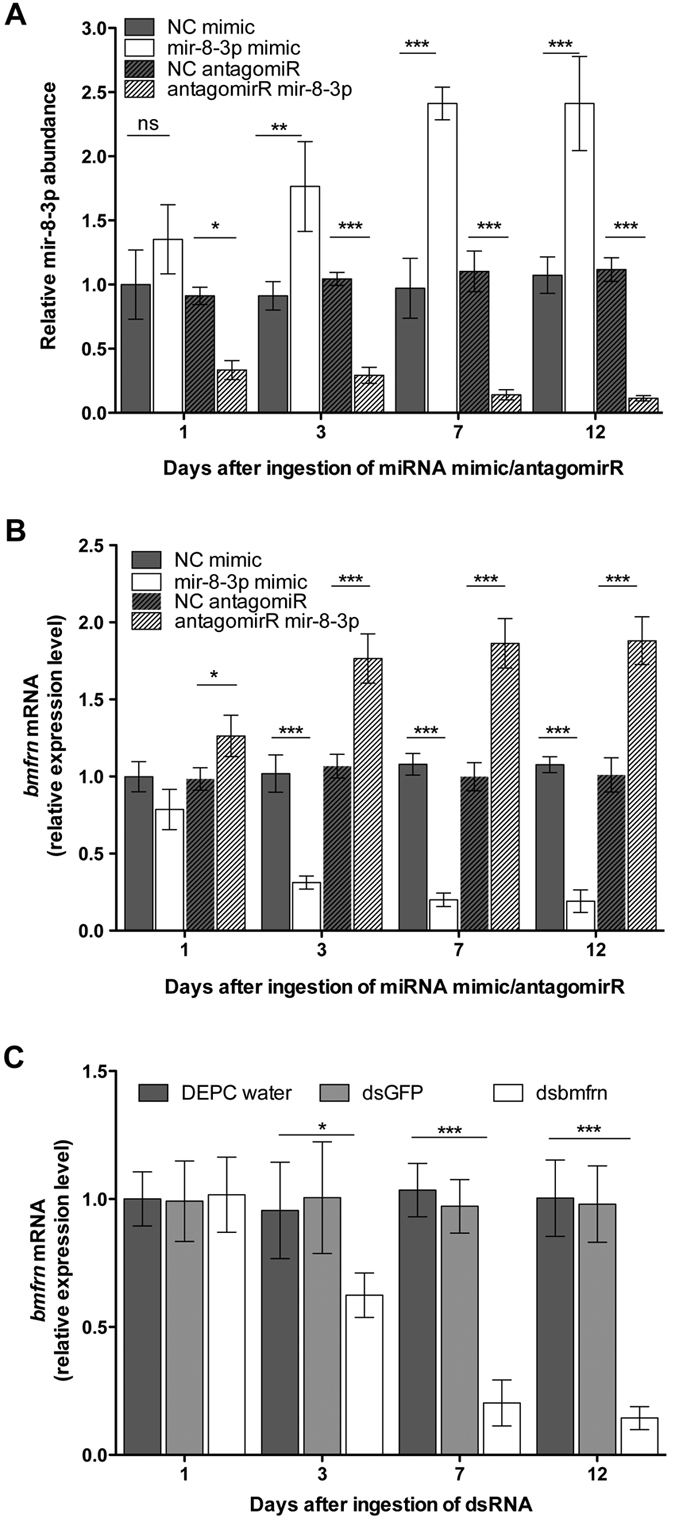
Dietary delivery of miR-8-3p mimics/antagomiRs and *bmfrn* dsRNA alter *bmfrn* expression in testes. Relative abundances of miR-8-3p (**A**) and *bmfrn* (**B,C**) were determined by qRT-PCR of cDNA made from total RNA isolated from testes 1, 3, 7, and 12 days after the indicated dietary treatments of adult flies. Data represent the mean values ± SD of three independent experiments (20 flies per experiment and treatment). Treatments were compared with their respective controls using ANOVA (t-test, p < 0.05).*, ** and *** indicates P < 0.5, P < 0.01 and P < 0.005 respectively.

To test the efficiency of diet-mediated delivery of amiR-8-3p mimic, amiR-8-3P antagomiR and *bmfrn* dsRNA (RNAi), we fed adult flies with a synthetic diet^23^ soaked with the respective treatments. The relative abundance of miR-8-3p and *bmfrn* mRNA was measured by qRT-PCR in testes after swapping flies for 1, 3, 7, and 12 days to the respective diets. The relative abundance of miR-8-3p was increased approximately two fold and significantly on days 3to 12of miR-8-3p mimic diet (Fig. 2A), resulting in a strong reduction (approximately 75%)of *bmfrn* expression in the same time frame (Fig. 2B). Treatment with the antagomiR, on the other hand, reduced the relative abundance of miR-8-3p on days 1–12 to about 30–12% of the control treatment (Fig. 2A) and increased the expression of *bmfrn* mRNA mildly but significantly on day 1 and about 2 fold on days 3–12 (Fig. 2B). RNAi treatment of flies with *bmfrn*dsRNA also resulted in a significant reduction of *bmfrn* mRNA expression from day 3 to 12 (Fig. 2C).

Comparison of the relative abundance of miR-8-3p and *bmfrn* mRNA of control treatments (NC) between days 1 to 12 indicates that they are not differentially expressed over time (Fig. 2A,B).With the confirmation that dietary delivery of miR-8-3p and *bmfrn*dsRNA successfully alter *bmfrn* expression in testis, we set out to functionally analyze the consequences of the above treatments on male reproductive capacity (relative number of successfully fertilized eggs), sperm number and sperm viability.

**Figure 3:**
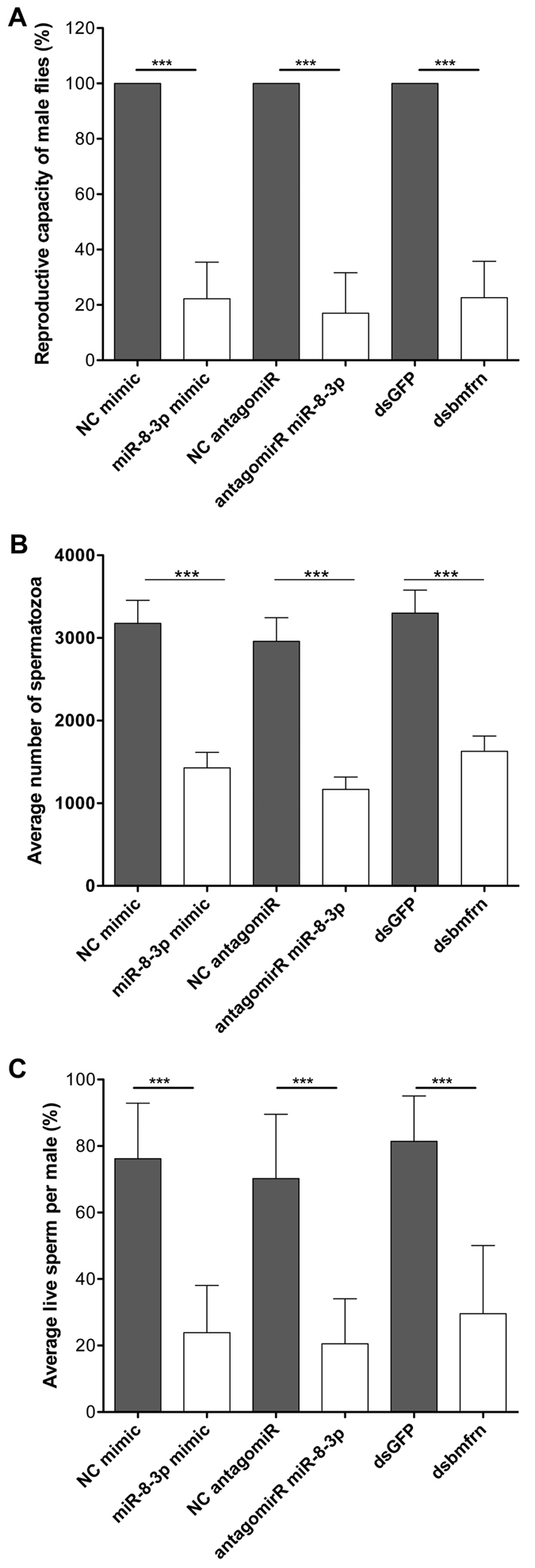
Increasing or decreasing miR-8-3p levels in testes, as well as reduction of *bmfrn* expression in the testis result in reduced reproductive capabilities due to reduced sperm production and viability. Newly eclosed male flies, maintained for 1, 3, 7 or 12 days on indicated treatments were used to determine (**A**) reproductive capacity, (**B**) sperm counts and (**C**) sperm viability. (**A**) Single males were mated with three females, eggs were collected and the percentage of larvae eclosed from the eggs was determined. Data represent the mean + /− SD from 20 males per treatment. (**B**) Spermatozoa from the seminal vesicles of individual males were counted. Data represent mean + /− SEM from 30 males per treatment. (**C**) Average percentage of live sperms per male was determined for seminal vesicles of 20 males per treatment. The effect of treatments was analyzed using generalized linear models with a binomial error distribution and a logit-link function ***indicates P < 0.0001.

We increased and reducedmiR-8-3p levels and silenced the *bmfrn* gene through RNAi in *B. dorsalis* male adult flies and quantified male fertility, sperm viability and total number of spermatozoa in seminal vesicles after 12 days of the respective treatment. We found significant reduction in the reproductive capacity (about 80% lower than that of controls) of male *B. dorsalis* flies treated with miR-8-3p mimic, miR-8-3p antagomiR and *bmfrn* dsRNA when compared to the respective controls (*X*^*2*^ = 22.25, df = 1, *P* < 0.0001; *X*^*2*^ = 17.24, df = 1, *P* < 0.0001; *X*^*2*^ = 22.60, df = 1, *P* < 0.0001 for miRNA mimic, antagomiR and RNAi treatments respectively, n = 20 per treatment)(Fig. 3A).

Reduced reproductive capacity of male flies can have several causes, such as altered mating behavior, malformed sexual organs or defects during spermatogenesis. As deletion of *mitoferrin* resulted in defects in spermatogenesis in *Drosophila melanogaster*^19^, we directly quantified the number of spermatozoa of male oriental fruit flies after 12 days of the above-mentioned treatments to investigate whether also in *B. dorsalis* spermatogenesis is affected by mitoferrin depletion. Compared to control males, the average number of sperm in the seminal vesicles ofmiR-8-3p mimic, miR-8-3p antagomiR and *bmfrn* dsRNA treated males were also significantly reduced by a factor of 2–3 (*X*^*2*^ = 380.06, df = 1, *P* < 0.0001; *X*^*2*^ = 493.4, df = 1, *P* < 0.0001; *X*^*2*^ = 865.03, df = 1, *P* < 0.0001; for miRNA mimic, antagomiR and RNAi treatments respectively, N = 30 per treatment)(Fig. 3B).

Since a sperm reduction by a factor of 2–3 would be unlikely to explain a drop in reproductive capacity to 20% of control animals, we suspected that not only the number of sperm was affected by the treatments, but also the viability of the spermatozoa. Therefore, we determined sperm viability and found a reduction of sperm viability to about 20% in treated male flies compared to their respective controls (*X*^*2*^ = 11.35, df = 1, *P* < 0.0001; *X*^*2*^ = 31.25, df = 1, *P* < 0.0001, *X*^*2*^ = 17.93, df = 2, *P* < 0.0001; for miRNA mimic, antagomiR and RNAi treatments respectively, n = 30 per treatment) (Fig. 3C). These results indicate, that the main reason for the reduced reproductive capacity of miR-8-3p mimic/antagomiR or *bmfrn* dsRNA treated male flies is the result of reduced sperm viability and not as much in the reduction in sperm number.

## Discussion

In this study, we provide evidence, for the first time, that a mitoferrin is regulated by a miRNA. Specifically, we show that miR-8-3p regulates *mitoferrin* (*bmfrn*) gene expression in the testes of *B. dorsalis* and is important for spermatogenesis. Recently, Calla and Geib reported that bdo-miR-8 is an miRNA conserved between *B. dorsalis* and *Drosophila*^24^. However, their analysis did not identify *bmfrn* as a target of bdo-mir-8. Indeed, also according to our binding-energy analysis (Fig. 1B), mir-8-3p was not the top-rating candidate, but turned out to be the only of the five miRNA candidates that showed a significant interaction with the 3′UTR of *bmfrn* mRNA (Fig. 3C), underscoring the importance of experimental validation of miRNAs and their targets. Of note, Calla and Geib did report dme-miR-315 as a potential miRNA targeting *dmfrn*^24^ and human mfnr1 and mfrn2 are annotated to potentially interact with at least seven miRNAs (www.microrna.org) indicating that miRNA-mediated regulation of *mitoferrins* might be a more broadly conserved mechanism. In the case of miR-8-3p, its tissue expression pattern suggests that it regulates mitoferrin expression in thorax and head. However, in flies treated with miR-8-3p or the antagomir we did not notice any obvious alterations in morphology or behavior connected to these tissues. Quantitative measures would probably be required to check for miR-8-3p associated effects in these body parts.

We observed that dietary delivery of miR-8-3p or RNAi resulted in reduction of *mitoferrin* expression in testes and reduction in sperm viability, total number of mature sperms in seminal vesicles and reproductive capacity of male flies. This phenotype is similar to hypomorph alleles in the *D. melanogaster mitoferrin* gene, which, in contrast to null-alleles, resulted in partial sterility only^19^. This can easily be explained by the fact that RNAi as well as miRNA treatments did not completely suppress *bmfrn* expression. In the case of miR-8-3p it cannot be excluded that also other genes than *mitoferrin* are targeted. However, reduction of *mitoferrin* expression in the testes by both, miR-8-3p mimic or *bmfrn* RNAi treatment to similar levels (20% of control respectively, day 7) led to similar reductions in spermatozoa viability and male reproductive capacity (sperm viability: 23% for miR-8-3p mimic and 30% for *bmfrn* RNAi; male reproductive capacity: 22% for miR-8-3p mimic and 22% for *bmfrn* RNAi). This would argue that *mitoferrin* is the main target of miR-8-3p and that any other targets affected by increased levels of miR-8-3p play a minor role in spermatogenesis. Interestingly and unexpectedly, we observed almost identical effects on male reproductive capacity, sperm viability and sperm counts, when miR-8-3p was inhibited by an antagomiR. As this treatment increased *mitoferrin* expression two-fold in the testis, it indicates that the tolerance of altered *mitoferrin* expression during spermatogenesis could be minimal.

In yeast it was shown biochemically that mitoferrins are required under iron-limiting conditions to provide sufficient mitochondrial iron for heme and Fe/S-cluster synthesis^18^. A similar function seems to be attributable to *Drosophila mitoferrin*, as deletion of *mitoferrin* is only lethal on low iron food^19^,^25^. Therefore, we would attribute the decreased sperm viability of miR-8-3p and *bmfrn* RNAi treated *B. dorsalis* mainly to insufficient heme and/or Fe/S cluster synthesis in spermatids as proposed for *Drosophila mitoferrin* loss of function mutants^19^. As heme and iron-sulfur clusers are co-factors of many different proteins, involved in diverse cellular functions, such as energy metabolism, P450 enzyme activity, hormone synthesis^25^, protein translation and many more^26^, any of these processes could be critically interfered with by insufficient mitochondrial iron supply.

The detrimental effect that miR-8-3p antagomiR has on sperm viability could have several reasons. Increased *mitoferrin* expression is likely to result in dysregulated cellular iron homeostasis, as shown for *Drosophila* cells^15^, where overexpression of *dmfrn* resulted in decreased total cellular iron levels and increased levels of *FerHCH* mRNA and protein. This in turn could negatively affect DNA synthesis as ribonucleotide reductase, a cytoplasmic enzyme^27^, requires iron ions as cofactors in its reactive center^28^,^29^. On the other hand, *mitoferrin* overexpression together with reduced levels of frataxin resulted in increased neurotoxicity and reduced mitochondrial functions in *Drosophila*^30^, indicating that iron transported by *mitoferrin* can have detrimental effects on mitochondria.

From our results it is not possible to identify the specific cause of reduced sperm viability and whether the defects in spermatogenesis or sperm function leading to reduced sperm viability and reproductive capacity are the same in flies treated with miR-8-3p mimic/antagomire or *bmfrn* dsRNA. However, our results clearly show that depleting or increasing miR-8-3p or RNAi of *mitoferrin* in *B. dorsalis* all lead to significantly reduced sperm viability and reduced male reproductive capacity.

## Materials and Methods

### Insects and cells

The oriental fruit flies (*Bactrocera dorsalis)* were maintained as previously reported^23^ at 27 ( ± 1) °C, under 14 :10 h light:dark photoperiod and 75 (±5)% relative humidity. Larvae were reared on a diet of bananas and adult flies were maintained on a diet consisting of yeast extract, sugar, honey, and agar.

HeLa (human cervical cancer) cells were cultured in Dulbecco’s Modified Eagle’s Medium (Gibco, Invitrogen) supplemented with 10% fetal bovine serum (Gibco CA), and 1% penicillin/streptomycin (Gibco, Invitrogen) at 37 °C in 5% CO_2_.

### Bioinformatics

Prediction of miRNAs targeting the 3′ UTR of the *bmfrn* gene was carried out by submitting the 3′-UTR sequence of the *mitoferrin* gene (Genbank number XM_011200779) and miRNA candidates to RNAhybrid^21^ (http://bibiserv.techfak.unibielefeld.de/rnahybrid/submission.html) using default parameters. Amino acid sequences of mitoferrins were aligned using CLC Main Workbench 6.9. with parameters: gap open cost 15.0, gap extrension cost 5.0, end gap cost “cheap”, alignment mode “very accurate (slow)”, redo alignments “yes” and use fixpoints “no”.

### miRNA mimics, antagomiRs and dsRNAs for cell culture and dietary treatments

Mimics of miR-279-3p, miR-8-3-p, miR-275-3p, miR-34-3p and miR-304-5p (sequences of these miRNAs are listed in Table S1) as well as a negative control (NC) were synthesized by GenePharma (Shanghai, China). The NC was based on a *Caenorhabditis elegans* miRNA (sequences are listed in Table S1) with no sequence similarity to known insect or mammalian miRNAs^31^.

For RNAi, a 320 bp long PCR product of *bmfrn* and a 495 bp long PCR product of the GFP gene^32^ (for primers see Table 1) served as templates to synthesize dsRNAs by the T7 RiboMAX™ Express RNAi System (Promega, US) followed by purification using the RNeasy MinElute Cleanup Kit (Qiagen, Germany). GFP dsRNA was used as a negative control. AntagomiRs and mimics were purchased from GenePharma (Shanghai, China). To increase the abundance of miR-8-3p, a miR-8-3p mimic and a NC mimic (unrelated mimic) were used. To silence the expression of miR-8-3p, an antagomiR (antagomiRmiR-8-3p) consisting of the reverse complement of miR-8-3p and for negative control a scrambled RNA (N.C antagomiR) were used^22^,^33^.

### Dual luciferase reporter (DLR) assay

The 3′-UTR of *bmfrn* gene (496-bp) was cloned from *B. dorsalis* testes (primers are listed in Table 1), inserted into 3′-end of the *Renilla* luciferase gene within the psiCheck-2 vector (Promega, USA) using NotI and XhoI sites. The psiCHECK-2 vector also contains a constitutively expressed firefly luciferase gene allowing normalization of renilla luciferase signals. For transfection, cells were seeded in 96-well plates at 10^4^ cells per well in serum-containing medium without antibiotics 12 h before transfection. The cells were transfected with 50 nM miRNA mimics or NC and 100 ng per well of the 3′ UTR luciferase reporter vector using 0.3 μL per well Lipofectamine 2000 (Invitrogen). 48 h after transfection, cells were lysed in 1X passive lysis buffer (Promega, USA) and activities of firefly and renilla luciferase were measured with the Dual-Luciferase Assay System (Promega, USA) according to the manufacturer’s protocol. Firefly luciferase values were normalized to renilla, and the ratio of firefly/renilla was presented.

### Dietary delivery of miRNA mimics, antagomiRs and dsRNAs to adult flies

Newly emerged male flies (within 12 h after eclosion) were collected. Flies were split into 7 groups of 140 individuals each to be used for the different treatments. Each treatment group was replicated three times. The adult diet was prepared using distilled water (UltraPure-Invitrogen) to avoid RNase activity and was equally split up for each treatment group. 400 μL of each treatment solution (miRNA mimic/antagomiR, dsRNA or solvent only) were added to 15 g of diet. dsRNA was added at 1 μg/μL^23^. Different concentrations of miRNA mimic or antagomiR have previously been tested and 100 nM of antagomiR miR-8-3p and 500 nM of miRNA mimic were found to be the most effective^22^,^34^. NCs were used at the same concentrations as the respective specific reagents. The diets with treatments were renewed every other day and lasted up to 12 days.

### RNA Isolation and Quantitative Real-Time PCR

Total RNAs were extracted from the testis of *B. dorsalis* using TRIzol reagent (Invitrogen, CA, USA) and cDNA was synthesized using 1 μg total RNA and miScript Hiflex buffer, in a final volume of 20 μL of miScript II reverse transcriptase reaction (Qiagen, Valencia, CA) according the manufacturer’s protocol. Modified oligo-dT primers with 3′ degenerate anchors and 5′ universal tag sequences were used for the specific synthesis of cDNA of mature miRNA and mRNA. miRNA primers (Table 1) for qPCR were designed using the miScript miRNA product design webpage (Qiagen, Valencia, CA). Primers for *bmfrn*, *actin* and *U6* cDNA (Table 1) were designed using Primer Premier 5.0 software (Premier, Canada). qRT-PCR was made up of 2.5 μL of 10x diluted cDNA, 12.5 μL of SYBR Green Master Mix (miScript SYBR Green PCR Kit, Qiagen Valencia, CA), 2.5 μL of 10 mM of forward and reverse primer and 5 μL of RNase free water in a 25 μL total volume. qRT-PCR was conducted on MyiQ2 real time PCR Detection System (BioRad) with the following thermal profile: 95 °C for 15 min, followed by 40 cycles of 94 °C for 15 s, 55 °C for 30 s and 70 °C for 30 s. Three technical replicates per sample were conducted and relative expression levels were calculated using 2^−△△Ct^ method^35^. Expression of *actin* and *U6* were used to normalized the expression of mRNA and miRNA respectively^14^,^36^

### Reproductive capacity of male flies

This assay was performed by crossing single male (from the treatments as indicated in results and figure captions) crossed to three 12 days old virgin females for two days. Eggs were collected^37^ within 4 hr of oviposition, transferred to Petri dishes containing banana pulp and incubated under controlled environmental conditions to permit embryonic development. After 5–7 days larvae were counted and reproductive capacity was calculated from the percentage of larvae per eggs.

### Sperm viability assays and spermatozoa counts

The seminal vesicles of each insect were carefully dissected in Hayes solution (9 g NaCl, 0.2 g CaCl_2_, 0.2 g KCl, and 0.1 g NaHCO_3_ in 1,000 ml H_2_O) and punctured with watchmaker forceps (Inox 5). A sample of 2 μl of out-flowing sperms were collected with a pipette and diluted in 250 μl of Hayes solution. Sperm viability was measured using a Live/Dead™ sperm viability kit (L-7011, Molecular Probes) using a previously developed protocol^38^,^39^. The kit consists of two fluorescent dyes that allow the experimenter to differentiate live (green emission, SYBR-14 dye) from dead sperm cells (red emission, propidium iodide).We first incubated 5 μl of diluted sperm with 5 μl of SYBR-14 working solution (2 μl SYBR-14 stock in 98 μl Hayes solution) on a glass microscope slide in the dark at 25 °C for 10 min, followed by 7 min incubation with propidiumiodide. To quantify sperm viability we used a UMNG2 filter (Olympus, Japan) microscope at 400 × magnifications and counted the number of live and dead cells for at least 400 sperm cells per slide. Dual-stained cells (max 1.6% per sample) of the total sperm population were excluded from the data. Sperm viability was calculated for each sample as the percentage of live sperm in the total number of sperm counted. To validate the experimental protocol, sperm were killed by freezing for 8 h at −80 °C. As expected all sperms in this sample stained red (dead).

Sperm counts were performed using the method described by^40^. In brief, sperm were extracted and spermatozoa were fixed in ethanol, air-dried and stained with DAPI for 15 min. Nuclei of individual spermatozoa were visualized and counted using a fluorescence microscope.

### Statistical analysis

The DLR assay and quantitative real-time PCR results were analyzed using one-way analysis of variance (ANOVA) with t-test or LSD using SPSS 19.0 for Windows software (SPSS Inc., Chicago). The level of significance was set at P < 0.05. We used SAS 9.2 for Windows (SAS Institute, Cary, NC) to analyze the overall effect of treatments on male fertility, sperm viability and total number of spermatozoa in seminal vesicles of male flies using a generalized linear model with a binomial error distribution and a logit-link function. The data were over-dispersed so we estimated the dispersion parameter from the scaled Pearson Chisquare. To examine differences between the treatments, pair-wise contrasts were used. *X*^2^ values are presented to show the treatment effects.

## Additional Information

**How to cite this article**: Tariq, K. *et al.* miR-8-3p regulates *mitoferrin* in the testes of *Bactrocera dorsalis* to ensure normal spermatogenesis. *Sci. Rep.* **6**, 22565; doi: 10.1038/srep22565 (2016).

## Supplementary Material

Supplementary Information

## Figures and Tables

**Table 1:** Primers used in this study.

Primers	Primer sequence
For cloning bmfrn-3UTR	
bmfrn-F	CGCTCGAGTTCAAAGAGTGATACTCAGAA
bmfrn-R	GCGGCCGCTCTTCTGTTTTTCTTCATTGAG
For dsRNA synthesis	
bmfrn-F	GGATCCTAATACGACTCACTATAGGAAAGTTTATCGCCAGTCAC
bmfrn-R	GGATCCTAATACGACTCACTATAGGACTTGGATGCCCTCAGTAC
dsGFP-F	GGATCCTAATACGACTCACTATAGGATACGGCGTGCAGTGCT
dsGFP-R	GGATCCTAATACGACTCACTATAGGATGATCGCGCTTCTCG
For qRT-PCR	
bmfrn-F	CTGCCTTCACAACGCCACT
bmfrn-R	GCCGTATTGCTTCACCCA
Actin-F	CGTTTCCGTTGCCCAGAATTCC
Actin-R	TCAGCAATACCTGGGTACATG
miR-8-3p	TAATACTGTCAGGTAAAGATGTC
U6	AGGATGACACGCAAAATCGT
