# Supplementary material for: miR-8-3p regulates mitoferrin in the testes of Bactrocera dorsalis to ensure normal spermatogenesis
Source: Sci Rep. 2016 Mar 2;6:22565. doi: 10.1038/srep22565 (PMC4773865; doi:10.1038/srep22565)
Supplement: Supplementary Information [file srep22565-s1.pdf]

Supplementary information

miR-8-3p regulates *mitoferrin* in the testes of *Bactrocera dorsalis* to ensure normal spermatogenesis.

KaleemTariq[a], Christoph Metzendorf[b], Wei Peng[a], Summar Sohail[a], Hongyu Zhang [a*]

[a]State Key Laboratory of Agricultural Microbiology, Hubei Key Laboratory of Insect Resource Application and Sustainable Pest Control, and Institute of Urban and Horticultural Entomology, College of Plant Science and Technology, Huazhong Agricultural University, Wuhan 430070, Hubei, People's Republic of China.

[b]Heidelberg University Biochemistry Center (BZH), University of Heidelberg, ImNeuenheimer Feld 328, Heidelberg, Germany.

* Author to whom correspondence should be addressed;

E-Mail: hongyu.zhang@mail.hzau.edu.cn; Tel.: +86-027-8728-0276; Fax: +86-027-8728-0796.

Supplementary Table

Table S1. miRNA mimics and antagomiRs used in this study.

Supplementary Figure

Figure S1: The putative *Bactrocera dorsalis* mitoferrin (bmfrn) amino acid sequence contains conserved histidine residues required for mitoferrin iron transport function. Amino acid sequences from mitoferrins of *B. dorsalis* (GI: 751779949, NCBI), fruit fly (*dmfrn*, flybase), yeast (GI: 1008338 and GI: 486507, NCBI) and human (GI: 82775373 and GI: 149274643, NCBI) were aligned using CLC Main Workbench 6.9 (CLC bio, Denmark) using the "very accurate"setting. Conserved histidine residues required for mitoferrin iron transport function[19] are indicated by asterisks.

miRNA	Sequence
**Mimics**	
miR-279-3p-sense strand	UGACUAGAUCCACACUCAUU
miR-279-3p-antisense strand	GAUGAGUGUGGAUCUAGUCA
miR-8-3-p-sense strand	UAAUACUGUCAGGUAAAGAUGUC
miR-8-3-p-antisense strand	ACAUCUUUACCUGACAGUAUUAU
miR-275-3p-sense strand	UCAGGUACCUGAAGUAGCGCGCG
miR-275-3p-antisense strand	CGCGCUACUUCAGGUACCUGAUU
miR-34-3p-sense strand	CAGCCACUAUCUUCACUGCCGCC
miR-34-3p-antisense strand	CGGCAGUGAAGAUAGUGGCUGUU
miR-304-5p-sense strand	UAAUCUCAAUUUGUAACUGUGA
miR-304-5p-antisense strand	ACAGUUACAAAUUGAGAUUAUU
N.C mimc-sense strand	UUCUCCGAACGUGUCACGUTT
N.C mimc-antisense strand	ACGUGACACGUUCGGAGAATT
**antagomiRs**	
miR-8-3-p	AGACAUCUUUACCUGACAGUAUUA
N.C antagomiR	CAGUACUUUUGUGUAGUACAA

Table S1. miRNA mimics and antagomiRs used in this study.

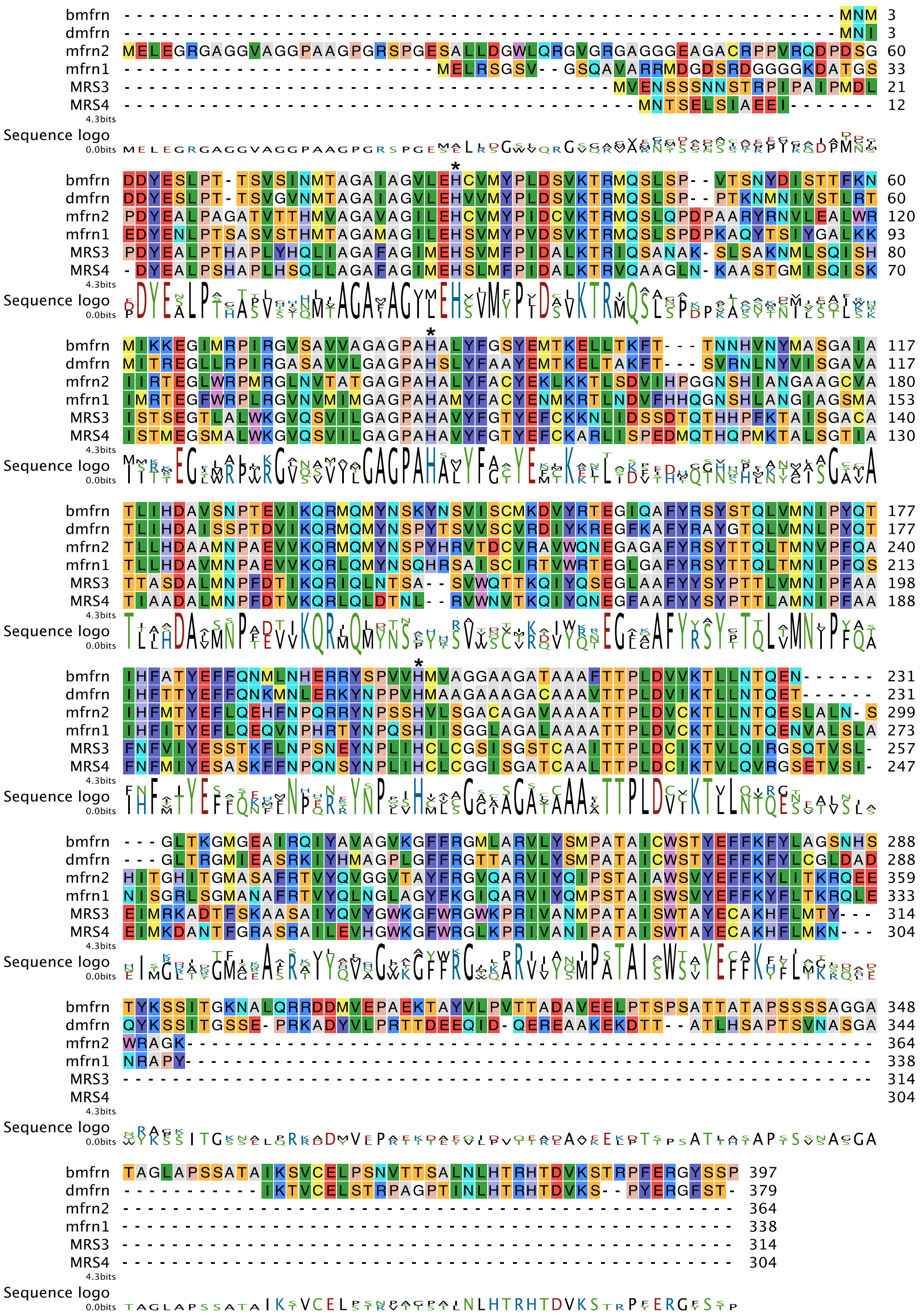

bmfrn ---MNM 3
dmfrn ---MNI 3
mfrn2 MELEGRGAGGVAGGPAAGPGRSPGESALLDGWLQRGVGRGAGGGEAGACRPPVRQDPDSG 60
mfrn1 -------------------------MELRSGSV--GSQAVARRMDGDSRDGGGGKDATGS 33
MRS3 ---------------------------------------MVENSSSNNSTRPIPAIPMDL 21
MRS4 ---MNTSELSIAEEI------- 12
Sequence logo
bmfrn DDYESLPT-TSVSINMTAGAIAGVLEHCVMYPLDSVKTRMQSLSP--VTSNYDISTTFKN 60
dmfrn DDYESLPT-TSVGVNMTAGAIAGVLEHVVMYPLDSVKTRMQSLSP--PTKNMNIVSTLRT 60
mfrn2 PDYEALPAGATVTTHMVAGAVAGILEHCVMYPIDCVKTRMQSLQPDPAARYRNVLEALWR 120
mfrn1 EDYENLPTSASVSTHMTAGAMAGILEHSVMYPVDSVKTRMQSLSPDPKAQYTSIYGALKK 93
MRS3 PDYEALPTHAPLYHQLIAGAFAGIMEHSVMFPIDALKTRIQSANAK-SLSAKNMLSQISH 80
MRS4 -DYEALPSHAPLHSQLLAGAFAGIMEHSLMFPIDALKTRVQAAGLN-KAASTGMISQISK 70
Sequence logo
bmfrn MIKKEGIMRPIRGVSAVVAGAGPAHALYFGSYEMTKELLTKFT---TNNHVNYMASGAIA 117
dmfrn MITREGLLRPIRGASAVVLGAGPAHSLYFAAYEMTKELTAKFT---SVRNLNYVISGAVA 117
mfrn2 IIRTEGLWRPMRGLNVTATGAGPAHALYFACYEKLKKTLSDVIHPGGNSHIANGAAGCVA 180
mfrn1 IMRTEGFWRPLRGVNVMIMGAGPAHAMYFACYENMKRTLNDVFHHQGNSHLANGIAGSMA 153
MRS3 ISTSEGTLALWKGVQSVILGAGPAHAVYFGTYEFCKKNLIDSSDTQTHHPFKTAISGACA 140
MRS4 ISTMEGSMALWKGVQSVILGAGPAHAVYFGTYEFCKARLISPEDMQTHQPMKTALSGTIA 130
Sequence logo
bmfrn TLIHDAVSNPTEVIKQRMQMYNSKYNSVISCMKDVYRTEGIQAFYRSYSTQLVMNIPYQT 177
dmfrn TLIHDAISSPTDVIKQRMQMYNSPYTSVVSCVRDIYKREGFKAFYRAYGTQLVMNLPYQT 177
mfrn2 TLLHDAAMNPAEVVKQRMQMYNSPYHRVTDCVRAVWQNEGAGAFYRSYTTQLTMNVPFQA 240
mfrn1 TLLHDAVMNPAEVVKQRLQMYNSQHRSAISCIRTVWRTEGLGAFYRSYTTQLTMNIPFQS 213
MRS3 TTASDALMNPFDTIKQRIQLNTSA--SVWQTTKQIYQSEGLAAFYYSYPTTLVMNIPFAA 198
MRS4 TIAADALMNPFDTVKQRLQLDTNL--RVWNVTKQIYQNEGFAAFYYSYPTTLAMNIPFAA 188
Sequence logo
bmfrn IHFATYEFFQNMLNHERRYSPVVHMVAGGAAGATAAAFTTPLDVVKTLLNTQEN------ 231
dmfrn IHFTTYEFFQNKMNLERKYNPPVHMAAGAAAGACAAAVTTPLDVIKTLLNTQET------ 231
mfrn2 IHFMTYEFLQEHFNPQRRYNPSSHVLSGACAGAVAAAATTPLDVCKTLLNTQESLALN-S 299
mfrn1 IHFITYEFLQEQVNPHRTYNPQSHIISGGLAGALAAAATTPLDVCKTLLNTQENVALSLA 273
MRS3 FNFVIYESSTKFLNPSNEYNPLIHCLCGSISGSTCAAITTPLDCIKTVLQIRGSQTVSL- 257
MRS4 FNFMIYESASKFFNPQNSYNPLIHCLCGGISGATCAALTTPLDCIKTVLQVRGSETVSI- 247
Sequence logo
bmfrn ---GLTKGMGEAIRQIYAVAGVKGFFRGMLARVLYSMPATAICWSTYEFFKFYLAGSNHS 288
dmfrn ---GLTRGMIEASRKIYHMAGPLGFFRGTTARVLYSMPATAICWSTYEFFKFYLCGLDAD 288
mfrn2 HITGHITGMASAFRTVYQVGGVTAYFRGVQARVIYQIPSTAIAWSVYEFFKYLITKRQEE 359
mfrn1 NISGRLSGMANAFRTVYQLNGLAGYFKGIQARVIYQMPSTAISWSVYEFFKYFLTKRQLE 333
MRS3 EIMRKADTFSKAASAIYQVYGWKGFWRGWKPRIVANMPATAISWTAYECAKHFLMTY--- 314
MRS4 EIMKDANTFGRASRAILEVHGWKGFWRGLKPRIVANIPATAISWTAYECAKHFLMKN--- 304
Sequence logo
bmfrn TYKSSITGKNALQRRDDMVEPAEKTAYVLPVTTADAVEELPTSPSATTATAPSSSSAGGA 348
dmfrn QYKSSITGSSE-PRKADYVLPRTTDEEQID-QEREAAKEKDTT--ATLHSAPTSVNASGA 344
mfrn2 WRAGK--- 364
mfrn1 NRAPY--- 338
MRS3 -- 314
MRS4 304
Sequence logo
bmfrn TAGLAPSSATAIKSVCELPSNVTTSALNLHTRHTDVKSTRPFERGYSSP 397
dmfrn -----------IKTVCELSTRPAGPTINLHTRHTDVKS--PYERGFST- 379
mfrn2 --- 364
mfrn1 --- 338
MRS3 --- 314
MRS4 --- 304
Sequence logo

Figure S1: The putative *Bactrocera dorsalis* mitoferrin (bmfrn) amino acid sequence contains conserved histidine residues required for mitoferrin iron transport function. Amino acid sequences from mitoferrins of *B. dorsalis* (GI: 751779949, NCBI), fruit fly (*dmfrn*, flybase), yeast (GI: 1008338 and GI: 486507, NCBI) and human (GI: 82775373 and GI: 149274643, NCBI) were aligned using CLC Main Workbench 6.9 (CLC bio, Denmark) using the "very accurate"setting. Conserved histidine residues required for mitoferrin iron transport function[19] are indicated by asterisks.
